# Continuous Stabilization and Carbonization of a Lignin–Cellulose Precursor to Carbon Fiber

**DOI:** 10.1021/acsomega.2c01806

**Published:** 2022-05-05

**Authors:** Andreas Bengtsson, Jenny Bengtsson, Kerstin Jedvert, Markus Kakkonen, Olli Tanhuanpää, Elisabet Brännvall, Maria Sedin

**Affiliations:** † Division Bioeconomy and Health, RISE Research Institutes of Sweden, P.O. Box 5604, SE-114 86 Stockholm, Sweden; ‡ Division Material and Production, RISE Research Institutes of Sweden, P.O. Box 104, SE-431 22 Mölndal, Sweden; § Fibrobotics OY, Korkeakoulunkatu 1, FI-33720 Tampere, Finland

## Abstract

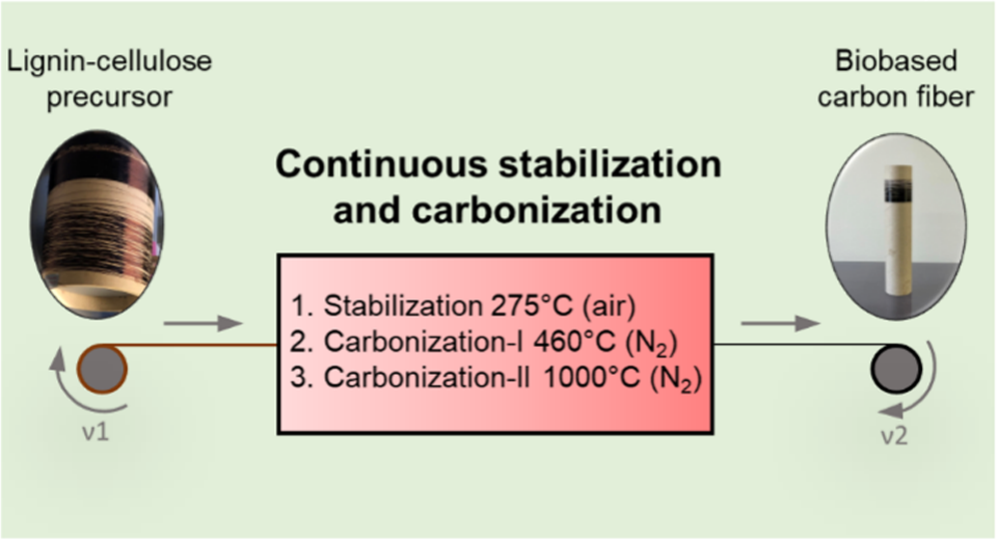

The demand for carbon fibers (CFs) based on renewable raw materials as the reinforcing fiber in composites for lightweight applications is growing. Lignin–cellulose precursor fibers (PFs) are a promising alternative, but so far, there is limited knowledge of how to continuously convert these PFs under industrial-like conditions into CFs. Continuous conversion is vital for the industrial production of CFs. In this work, we have compared the continuous conversion of lignin–cellulose PFs (50 wt % softwood kraft lignin and 50 wt % dissolving-grade kraft pulp) with batchwise conversion. The PFs were successfully stabilized and carbonized continuously over a total time of 1.0–1.5 h, comparable to the industrial production of CFs from polyacrylonitrile. CFs derived continuously at 1000 °C with a relative stretch of −10% (fiber contraction) had a conversion yield of 29 wt %, a diameter of 12–15 μm, a Young’s modulus of 46–51 GPa, and a tensile strength of 710–920 MPa. In comparison, CFs obtained at 1000 °C via batchwise conversion (12–15 μm diameter) with a relative stretch of 0% and a conversion time of 7 h (due to the low heating and cooling rates) had a higher conversion yield of 34 wt %, a higher Young’s modulus (63–67 GPa) but a similar tensile strength (800–920 MPa). This suggests that the Young’s modulus can be improved by the optimization of the fiber tension, residence time, and temperature profile during continuous conversion, while a higher tensile strength can be achieved by reducing the fiber diameter as it minimizes the risk of critical defects.

## Introduction

Due to their high specific stiffness and strength, commercial carbon fibers (CFs) are highly attractive as the load-bearing constituent in composites for structural applications. At present, there is an increasing demand for CFs in, e.g., automotive parts and wind turbine blades. The wider use of CFs is inhibited by their high price, owing to the use of the expensive fossil-based polymer polyacrylonitrile (PAN, >96%) and the energy-intensive production process, where the PAN precursor fiber (PF) accounts for about half of the total production cost.^1,2^

Lignin and cellulose are two renewables readily available in large quantities from, e.g., the kraft pulping process, and they are potential raw materials for biobased CFs. However, CF preparation from lignin or cellulose separately is challenging. Cellulose-based CFs were developed in the 1960s and the following 1970s. Later, PAN-based CFs were found to have several advantages; cellulose-based CFs are expensive due to the low carbon yield (10–30 wt %), which originates from the low carbon content of cellulose (44.4 wt %).^3,4^ Nevertheless, cellulose has a beneficial molecular orientation that makes it possible to obtain CFs with a high Young’s modulus (up to ∼500 GPa) after hot stretching above 2000 °C.^4−7^ The most favorable characteristics of kraft lignin are its high carbon content (60–65 wt %) and availability, but CFs made from melt-spun lignin PFs generally require very long stabilization times, sometimes over 100 h, making industrial production not feasible.^8,9^ Furthermore, for a successful melt spinning of lignin, the thermal properties of lignin are very important, and pretreatments such as solvent extraction and membrane filtration of the lignin is often necessary. To overcome this challenge, lignin has been derivatized and/or coprocessed with other polymers.^10−12^ One promising approach has been solvent fractionation of softwood kraft lignin in combination with a different spinning technique, dry spinning.^13^ Using batchwise conversion (a few mg) and a conversion time of 3.4 h, the authors obtained CFs with a Young’s modulus and tensile strength of 98 and 1.39 GPa, respectively.^13^ However, the need for solvent fractionation increases the cost of the CF. Coprocessing of lignin–cellulose blends into PFs via dry-jet wet spinning is also promising for various reasons.^14−20^ The stabilization time can be significantly reduced (<2 h) compared to that for melt-spun lignin fibers, and the CF yield after conversion is significantly higher than obtained with neat cellulose.^16,17,21^ The choice of dry-jet wet spinning (solution spinning) instead of melt spinning broadens the operation window with respect to the thermal properties of the lignin. In addition, the cellulose content makes the PF flexible and easy to handle, which is beneficial in CF preparation.

Most previous studies of the conversion of lignin and cellulose to CF have used a static batchwise conversion system. This setup is satisfactory in the early development work as it requires only small amounts of PF and is therefore suitable for studying the fundamental conversion behavior, but these furnaces are usually limited to low heating and cooling rates (typically ≤10 °C/min), leading to very long conversion times (>5 h) compared to typical conventional continuous conversion of 2 h for PAN-based CFs.^18^

In continuous conversion, the PF is passed through a series of stabilization (air or oxygen) and carbonization (inert) furnaces with a gradually increasing temperature and the carbon content in the fiber is increased to over 90 wt % by removal of heteroatoms such as oxygen, hydrogen, and nitrogen.^4^ Meanwhile, the fibers are subjected to tension, which retains or increases the molecular orientation, finally giving a stiff and strong CF on a bobbin. Suitable process conditions are a trade-off between maximizing the yield and mechanical properties while minimizing processing costs (mainly energy consumption). The optimal conditions in each conversion step depend on the PF and desired properties of the CF, but stabilization is generally the most time-consuming step irrespective of PF.^4,22^ In contrast to batchwise conversion, continuous conversion makes it possible to have higher heating and cooling rates and tension applied at specific temperatures. While the production of commercial CF from PAN can now be considered very mature with suitable conversion conditions (temperature profile, residence time, tension, gas flow, etc.), the opposite is true regarding the knowledge of how to convert lignin–cellulose PFs via continuous conversion, and this is thus the main motivation of this work. To the best knowledge of the authors, only one work in the open literature by Le and co-workers deals with continuous conversion of lignin–cellulose PFs (hardwood organosolv lignin and dissolving-grade hardwood kraft pulp).^19^ After continuous stabilization for 92 min at 240–270 °C and the subsequent carbonization at 800 °C for 5.5 min, they obtained hollow CFs with a Young’s modulus and tensile strength of about 27 GPa and 470 MPa, respectively. After carbonization at 1500 °C for 5.5 min, the authors reported that the CFs were fused to the extent that no CFs could be separated for tensile testing. The authors attributed the formation of the hollow CF morphology to an unstabilized core.

The aim of the present work is to study the continuous conversion of dry-jet wet spun lignin–cellulose PFs (softwood kraft lignin and dissolving-grade softwood kraft pulp) into CFs at industrially relevant process conditions and to compare the results with batchwise conversion. The choice of softwood kraft lignin instead of a hardwood lignin is due to the higher thermal reactivity of the former, which reduces the risk of fiber fusion and is beneficial for the reduction of the stabilization time.^23,24^

## Experimental Section

### Materials

Softwood kraft lignin produced by the LignoBoost process was obtained from LignoDemo (Bäckhammar, Sweden). A softwood dissolving-grade kraft pulp from Georgia Pacific (Atlanta, Georgia) was used as cellulose source. The solvent 1-ethyl-3-methylimidazolium acetate (EMIMAc, Aldrich 95%), was used as received. Further details regarding the raw materials can be found in our previous work.^16^

### Spinning of PFs

Prior to dissolution, the kraft lignin was sieved (0.5 mm), and the cellulose was ground (1 mm). The lignin was dried at 60 °C, and the cellulose was dried at 40 °C. Thereafter, equal amounts of lignin and cellulose (dry weight) at a total concentration of 16 wt % were simultaneously dissolved in EMIMAc at 70 °C for 1 h in a closed reactor with overhead stirring. Prior to spinning, the solution was deaerated at 60 °C in vacuum (<10 kPa) for at least 5 h.

The spinning equipment included a piston pump, a spin bath, and take-up rolls. The solution was extruded at 60 °C at 4 m/min through a die consisting of 33 capillaries with diameters of 120 μm. The solution passed through a 1 cm air gap before coagulation in deionized water. For fibers with a draw ratio (DR) of 2 (a take-up speed twice the extrusion speed), countercurrent washing steps and drying were performed in-line, including two wash steps (deionized water, RT, total residence time approximately 1.5 min) and a step for spin finishing (Neutral, Unilever, RT, 1 min) and hot air drying (60 °C, 2 min) before winding onto a bobbin. For the higher DR (DR4), due to the higher take-up speed, the coagulated fibers were first collected and then led through the washing steps, dryer and finally wound onto a bobbin. The PF tensile properties are summarized in Table S1 while a thorough investigation of the rheological behavior of the spinning solutions and microstructural characterization of the spun PFs can be found in our previous works.^25,26^

### Stabilization and Carbonization

**Figure 1:**
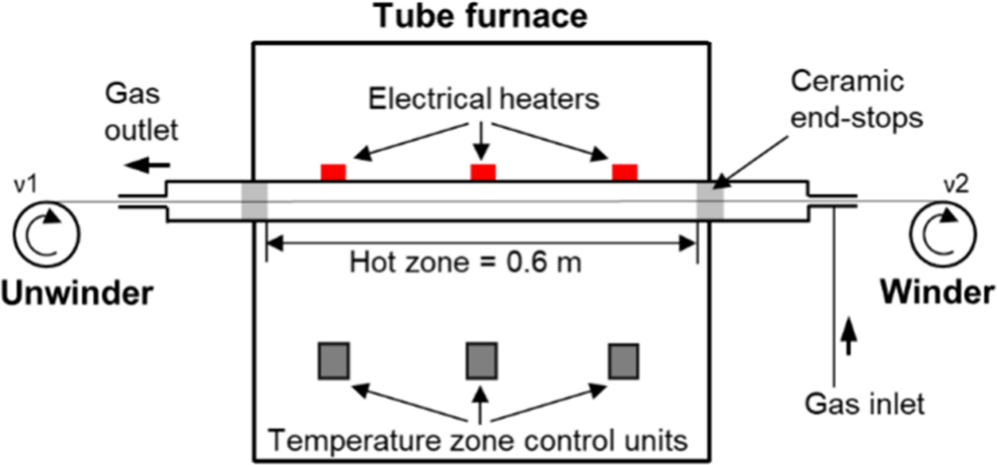
Schematic of the experimental setup used for continuous stabilization and carbonization.

The PFs were subjected to continuous stabilization (air) and carbonization (nitrogen) with a double winding system (Xplore Instruments BV, Netherlands) and a horizontal quartz tube furnace (OTF 1200x, MTI Corporation). The hot zone of the tube furnace was 0.6 m, with three programmable heating zones of about 0.2 m each. The volume of the heated part of the tube was 1.4 L, and the maximum furnace temperature employed was 1000 °C. The gas flow was set to 4 L/min and connected to the furnace to allow for an end-to-end flow countercurrent to the transport direction of the fiber tow (Figure 1).

The furnace temperature was calibrated with a thermocouple (CL3515R, Omega Engineering Inc.). The minimum operating speed of the winding unit was 10 mm/min, giving a maximum residence time in the furnace of 60 min. The relative stretch (%) of the fiber tow was adjusted by varying the relative speed of the unwinder and winder. A positive relative stretch indicates elongation of the fibers, whereas a negative value indicates fiber shrinkage. The studied conditions are summarized in Table 1. The outlet temperature (zone 3) in each step is used throughout the text to denote the profiles in Table 1. The PFs were first stabilized in air followed by reprogramming of the furnace and switch to nitrogen gas to carbonize the stabilized PFs in two steps, according to Table 1. For comparison, DR2 and DR4 PFs were converted into CFs using the batchwise conversion equipment used in our previous work.^18^ In short, the PFs were mounted on a graphite frame to prevent fiber shrinkage (relative stretch 0%) and then stabilized in a muffle furnace (air, 7 L/min) by heating from 25 to 250 °C at 5 °C/min and then held isothermally for 10 min, followed by a ramp to 260 and 275 °C using the same heating rate and an isothermal time at each temperature. The sum of the heating time and the isothermals gave a stabilization time of 79 min. This profile was chosen to mimic continuous stabilization using a 30 min residence time. Batchwise carbonization of the stabilized PFs was carried out in a tube furnace by heating at 3 °C/min from 25 to 1000 °C, followed by an isothermal of 17 min prior to the natural cooling down of the furnace, giving a carbonization time of 342 min.

**Table 1:** Summary of the Studied Conditions during Continuous Conversion^a^

	furnace temperature (°C)					
conversion step	zone 1	zone 2	zone 3	gas	relative stretch (%)	time (min)
stabilization	250	260	275	air	0	30, 45, or 60
carbonization–I	350	410	460	nitrogen	0	15
carbonization–IIa	800	800	800	nitrogen	–10	17
carbonization–Iib	1000	1000	1000	nitrogen	–10	17

a Zone 1: inlet; zone 2: middle; and zone 3: outlet in the direction of the fiber.

### Characterization

The mass yield of the CFs prepared via continuous conversion was estimated by measuring the linear density (expressed as dtex) after each conversion step and dividing that by the linear density of the PF. The linear density was measured by weighing 40–100 cm fiber tow. The gravimetric CF yield of the batchwise derived CFs was determined using about 75 mg of PF that was stabilized and carbonized without tension in a ceramic crucible. Because of the different ways of measuring the yield, the results may differ slightly.

The elemental composition (wt %) of the heat-treated fibers and CFs was estimated by energy-dispersive X-ray analysis (EDXA) using an Xflash detector (Bruker Corp.) at an acceleration voltage of 15 kV and the Esprit software for data evaluation. CHN analysis of the PFs (DR2 and DR4) was carried out in a LECO CHN 628 elemental analyzer (LECO) according to SS-EN-ISO 16948. The carbon yield after a specific conversion step was estimated by multiplying the mass yield by the carbon content and dividing that by the initial carbon content in the PF.

Single-fiber tensile tests were performed on a LEX820/LDS0200 (Dia-Stron Ltd., U.K.) equipped with a laser diffraction system for diameter determination (CERSA-MCI, France). The fibers were tested at a fixed gauge length of 20 mm at an elongation speed of 5 mm/min except for the CFs prepared at 800 and 1000 °C, which were tested at 0.6 mm/min. The data were evaluated with the UvWin software (Dia-stron Ltd., U.K.). The reported values for each sample are averages of 30–40 individual filament measurements. The free shrinkage (%) of the PF upon conversion was estimated by placing three strands of PF tow (20 cm length) with both ends free on a graphite support and allowing it to shrink during stabilization and carbonization.

Raman spectroscopy was performed with a 532 nm excitation laser (WITec Alpha 300 RAS, Witec, Ulm, Germany) on CF surfaces at three separate positions and on three individual filaments per sample. To ensure that the samples were not changed due to the laser, the laser power was kept at 2.0 mW. For detailed studies of the D and G bands, high-resolution spectra were collected at 1800 g/mm, centered at 1450 cm^–1^. Overview spectra were collected at 600 g/mm and centered at 2050 cm^–1^. The spectra were evaluated using the WITec Project 5.1 plus software (WITec, Ulm, Germany). The spectra were corrected for cosmic rays, and the background was subtracted by applying a shape-based correction with a diameter of 500 cm^–1^. The D and G bands were fitted applying a Lorentzian fitting within the WITec Project software.

The appearance of the PF and the CFs was evaluated in an SU3500 electron microscope (Hitachi, Japan) at an acceleration voltage of 3 kV using a secondary electron (SE) detector. Prior to imaging, the fibers were Ag-coated and then fixed on a sample holder using carbon tape. Cross sections were prepared by slitting the fibers with a scalpel.

Fourier transform infrared (FTIR) spectra (4000–650 cm^–1^) of PFs and stabilized PFs were recorded on a Varian 680-IR FTIR spectrometer equipped with an attenuated total reflectance accessory (ZnSe crystal). A total of 32 scans were captured at a spectral resolution of 4 cm^–1^ and subjected to baseline correction. The reported spectra are an average of three measurements.

A Q5000IR thermogravimetric analyzer (TGA) from TA Instruments was used to examine the thermal behavior of the PFs during oxidative stabilization (air, 25 mL/min) and carbonization (nitrogen, 25 mL/min). The PFs were chopped to a length of 2–5 mm, and 3.0 ± 1.0 mg of sample was placed in a platinum crucible. To mimic the stabilization, the samples were heated at 10 °C/min from room temperature to 250 °C, then to 260 °C, and finally to 275 °C. The samples were held at each temperature for 5, 10, or 15 min to mimic the conditions in Table 1. The stabilized PFs were then carbonized in a second TGA run by heating at 10 °C/min from room temperature to 350 °C and then 410 °C, followed by 460 °C (the samples being held for 5 min at each temperature) and then finally to 1000 °C at the same heating rate followed by 17 min at this temperature prior to cooling. The data were analyzed with the software Universal Analysis 2000 (TA Instruments).

Interfacial shear strength (IFSS) samples were prepared and the CF and epoxy resin (Epikote 135 and Epikure 137, Hexion) were measured with FIBRODrop and FIBROBond (Fibrobotics Oy, Finland) devices, respectively. Lignin–cellulose-derived CFs (DR2, 30 min stabilization time) were compared to a reference, which was a commercial T700S PAN-based CF (Toray Industries Inc., Japan). Prior to the measurements, the sizing of the reference CF was removed in a KSL-1200X muffle furnace (MTI Corporation) at 380 °C for 20 min in air (7 L/min). The droplet samples were cured for one day at room temperature, followed by 10 h at 40 °C. In the IFSS measurements, about 30 droplets with different sizes (25–104 μm in diameter) were measured on three parallel samples from each fiber. A detailed description of the devices and the analysis methods are given elsewhere.^27^

## Results and Discussion

In the manufacture of CFs, parameters such as temperature, time, gas flow rate, tensional load, and tow size may influence the mass yield and the tensile properties of the final CF. This section is structured into three main parts. The first part deals with the effect of temperature (treatment step) during continuous conversion and its impact on, e.g., the mass yield, elemental composition, and tensile properties, keeping the stabilization time constant (30 min). Here, carbonization temperatures up to 1000 °C and a total conversion time of 62 min were used, see Table 1. Since stabilization is the most time-consuming step in CF manufacturing, the second part focuses on the effect of stabilization time (30–60 min) on the tensile properties and yield of the CF. Finally, the third part presents an investigation of the interfacial adhesion (IFSS measurements) between epoxy resin and lignin–cellulose-derived CFs, which is an important aspect to consider in the development of CF reinforced composites.

### Change in Mass Yield and Elemental Composition during Continuous Conversion

In CF production, the mass yield has an impact on the process economy and is therefore important to maximize. It is therefore beneficial to use a PF with high carbon content such as softwood kraft lignin instead of cellulose since these contain 64 and 44 wt % carbon, respectively.^16^ During the conversion, the fiber composition gradually changes as the temperature increases, and a carbon content of at least 90 wt % in the fiber is usually required for it to be considered a CF.

**Figure 2:**
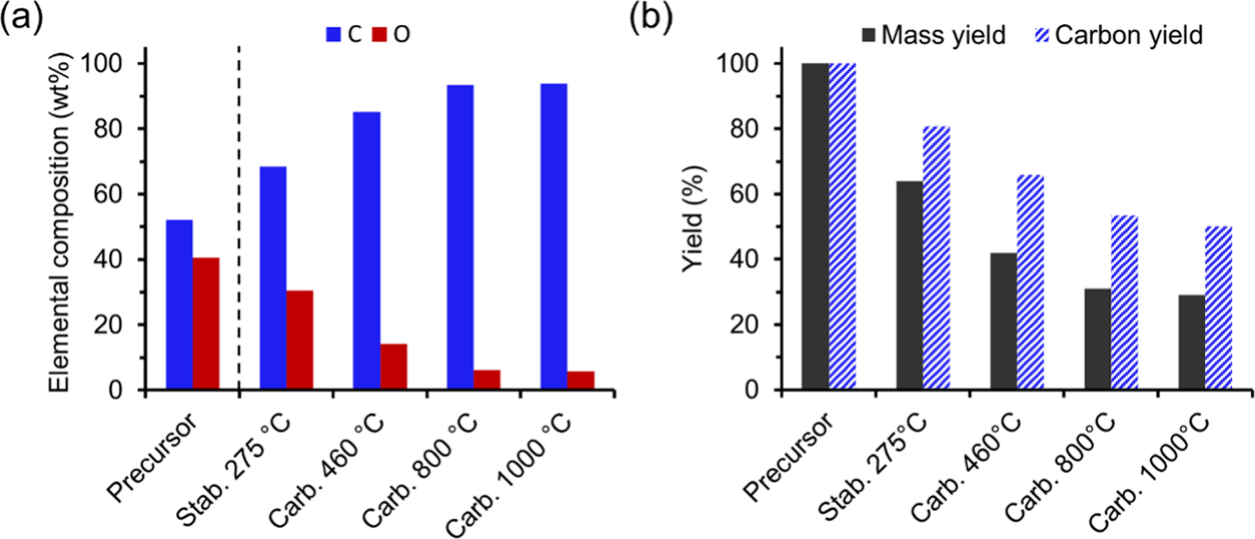
Effect of treatment temperature on (a) carbon and oxygen content and (b) the mass and carbon yield during the preparation of carbon fiber (CF) via continuous conversion of the DR2 lignin–cellulose precursor fiber (PF). The elemental composition of the PF was analyzed with CHN analysis, and the heat-treated fibers were analyzed with SEM-EDXA.

Initial attempts involved the direct carbonization of stabilized DR2 PFs (275 °C) at 800 or 1000 °C, but an intermediate low-temperature carbonization at 460 °C reduced the risk of filament breaks during the carbonization at 800 or 1000 °C. This may be due to the mass loss that occurred at 460 °C (Figure 2a), probably leading to less fiber stress in the subsequent high-temperature carbonization. This agrees with earlier observations during the continuous conversion of cellulosic rayon yarn into CF.^6^ Irrespective of rayon type, Strong observed that these fibers should be stabilized to a yield of 45–50 wt % to be effectively processed to CF at higher temperatures.^6^ X-ray diffraction revealed that the crystalline structure of cellulose completely disappeared at this mass yield, and thus the material was ready for further heat treatment. Figure 2a shows that regardless of carbonization temperature (800 or 1000 °C), no significant difference in carbon content (93–94 wt %) and oxygen content (6 wt %) was observed between the CFs, in agreement with our previous report on the effect of carbonization temperature during batchwise conversion of lignin–cellulose PFs.^18^ The yield after carbonization at 800 °C was 30 wt %, whereas a temperature of 1000 °C gave a yield of 29 wt %, suggesting that most of the mass loss occurs up to 800 °C (Figure 2b). The gravimetric yield for the CFs made via batchwise conversion was 34 wt %, i.e., higher than obtained in the continuous trials. In addition, Figure 2b shows the estimated carbon yield, which relates the carbon content and the mass yield after a specific thermal treatment to the carbon content of the PF (54 wt %). For example, the mass yield after oxidative stabilization was 64 wt %, and during this treatment, the carbon content increased to 68 wt % in the stabilized PF, giving a carbon yield of about 80%. After carbonization at 800 or 1000 °C, the carbon yield was about 50%, suggesting that half of the carbon present in the PF was retained in the CF after the thermal conversion.

The measured yield in the present work, 29–30 wt %, is significantly lower than what we previously observed during batchwise conversion (38–40 wt %).^17^ The reason for this is 2-fold. First, the PF in the present work was made from a 50/50 lignin/cellulose blend (wt/wt), while 70/30 blends were used previously.^16,17^ The higher carbon content of softwood kraft lignin is beneficial for the yield, and it is therefore expected that the conversion yield will be lower when starting with a lower fraction of softwood kraft lignin in the PF. On the other hand, a larger cellulose fraction in the PF makes the fibers stronger and easier to handle. Second, a higher stabilization temperature, 275 °C instead of 250 °C, was used in this study because cellulose has a higher thermal stability than softwood kraft lignin, and the former thus requires a higher temperature to be stabilized in a realistic residence time.^21^ In addition, the difference in total conversion time may have had an impact on the mass yield, as the slow heating and cooling rates employed in a batchwise setup lead to significantly longer stabilization and carbonization times than in continuous conversion.

### Effect of Temperature on the Tensile Properties during Continuous Conversion

**Figure 3:**
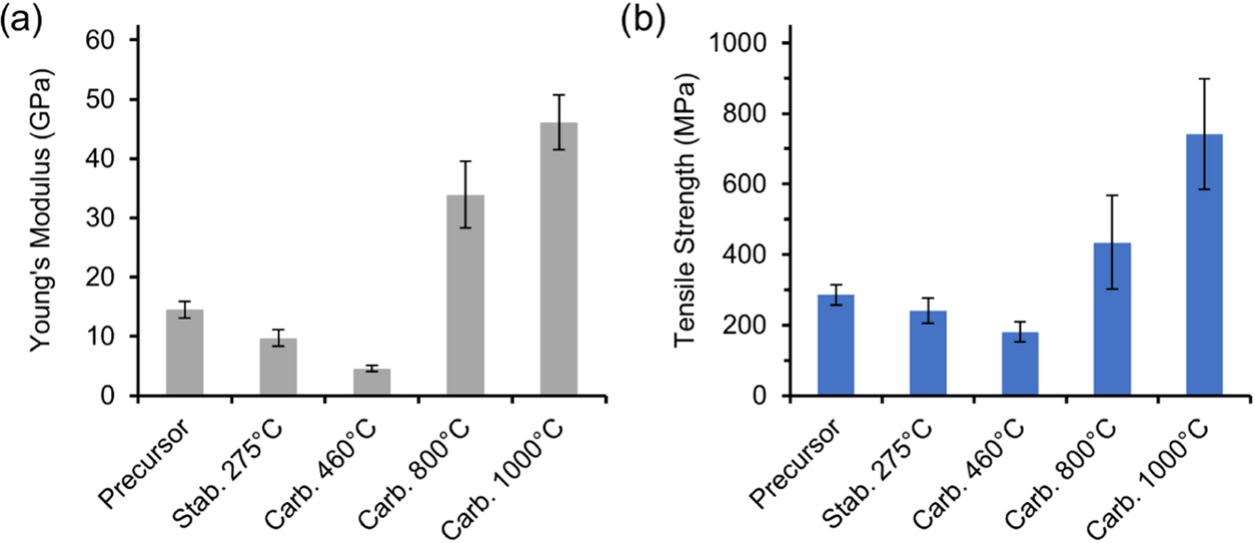
Effect of treatment temperature on (a) Young’s modulus and (b) tensile strength during the preparation of CF via continuous conversion of the DR2 lignin–cellulose PF. The error bars show the standard deviation.

After the lignin–cellulose PF (DR2) had passed stabilization (275 °C) and carbonization (460 and 1000 °C), the elongation at break was reduced from 5.5 to 1.6%, illustrative of its transformation into a brittle CF (Figure S1). Figure 3 shows the tensile properties of the PF and of the treated fibers after various treatment temperatures. The Young’s modulus and tensile strength displayed a similar behavior, a lowering of the tensile properties after stabilization and low-temperature carbonization at 460 °C, and thereafter a significant increase. After carbonization at 1000 °C, the CFs (diameter 14.5 μm) had a Young’s modulus and tensile strength of 46 GPa and 740 MPa, respectively. The same relationship between tensile properties and process temperature has been observed in the preparation of CFs from viscose fibers, suggesting that cellulose is important for the tensile properties in the conversion of lignin–cellulose PFs (50/50 wt/wt) into CF.^28^ The decrease in tensile properties during the stabilization and low-temperature carbonization at 460 °C is due to cellulose depolymerization and the loss of its crystalline structure.^6,29,30^ In contrast, the increase in the tensile properties after carbonization at 800 or 1000 °C is a result of the carbonization (removal of heteroatoms) and the formation of an amorphous (turbostratic) carbon structure.^18,19^

The CFs derived at 1000 °C had significantly higher tensile properties than those derived at 800 °C, in agreement with our earlier observations during batchwise conversion.^18^ This difference in fiber stiffness and strength may be due to a reorganization of the carbon structure at higher temperatures, such as an increase in crystallite size growth or a change in orientation. The crystallite size of graphitic domains is known to increase with increasing carbonization temperature, and this increases Young’s modulus.^31^

For comparison, CFs were prepared using the batchwise conversion setup under similar conditions (30 min stabilization, carbonization temperature 1000 °C). These CFs (diameter 15.3 μm) had a significantly higher Young’s modulus (63 GPa) but a similar tensile strength (800 MPa), see Table S2. This higher Young’s modulus may be due to the longer conversion time of 421 min in batchwise conversion, in contrast to the 62 min in continuous conversion. In addition, the fiber tension, which is known to have an impact on Young’s modulus of cellulose-based CFs, may have differed.^32,33^ To investigate possible differences in the carbon structure of the CFs derived via batchwise or continuous conversion, Raman spectroscopy was employed (Figure S2). The Raman spectra were almost identical, suggesting that the batchwise and continuously derived CFs had a similar carbon structure. Overall, the results are very similar to our previous Raman investigation on batchwise derived CFs at 1000 °C using a 70/30 wt/wt lignin–cellulose PF.^18^ Irrespective of conversion mode, the CFs had a similar intensity ratio of the D (1342 cm^–1^) and G (1590 cm^–1^) bands (*I*_D_/*I*_G_ = 0.9) and no significant difference in full width at half maximum of the D and G band could be observed (Table S3). The *I*_D_/*I*_G_ ratio is also in good agreement with other Raman measurements on lignin–cellulose-derived CFs.^15^ According to the famous three-stage model for disordered carbons proposed by Ferrari and Robertson, an *I*_D_/*I*_G_ of 0.9 suggests that amorphous carbon dominates the CF structure.^34^ Conclusively, the results suggest that Raman spectroscopy is not capable of explaining the significant difference in Young’s modulus of the continuously derived CFs (46 GPa) and the batchwise derived CFs (63 GPa).

In the stabilization (275 °C) and low-temperature carbonization (460 °C), it was possible to have a relative stretch of 0%, i.e., the same speed of the unwinder and of the winder, but a relative stretch of −10% was beneficial upon processing at 800 and 1000 °C. After carbonization at 1000 °C, the inherent free shrinkage in the longitudinal fiber direction was estimated to be 22%, which is comparable to the free shrinkage reported for carbonized melt-spun lignin fibers and man-made cellulose fibers.^35,36^ The inherent shrinkage was greater than the relative stretch (−10%), suggesting that the fibers were effectively stretched during conversion. The fibers could withstand a relative stretch of up to 50% in the low-temperature carbonization step, but as these experiments were controlled by relative speed instead of fiber tension, filament breaks occurred. In the future, improved CF tensile properties will be addressed using a tensiometer to optimize the fiber tension during conversion.

A reduction in fiber diameter is beneficial for the tensile properties of CFs as it reduces the probability of critical defects.^2,17^ Therefore, continuous conversion of the thinner PFs (DR4) was also carried out. The fiber diameter and tensile properties of the DR4 PFs are summarized in Table S1. Using the same conditions as for the DR2 fibers (30 min stabilization), a Young’s modulus and tensile strength of 49 GPa and 840 MPa, respectively, were obtained. These CFs had a diameter of 11.9 μm. In the same manner as for the DR2 PF, the DR4 PF was also converted to CF via batchwise conversion using a carbonization temperature of 1000 °C. These CFs (diameter 12.0 μm) had a Young’s modulus of 67 GPa and a tensile strength of 920 MPa (Table S2). This shows that regardless of DR, the CFs made via batchwise conversion obtained a significantly higher Young’s modulus than the continuously derived CFs and that the DR4 PFs showed slightly higher tensile properties than when made from the DR2 PFs, in agreement with our earlier work on batchwise conversion of lignin–cellulose PFs to CFs.^17^ In our previous work, we prepared CFs via batchwise conversion using lignin–cellulose PFs (70/30 wt/wt) spun with a DR of 7. These CFs had a diameter of 6.4–7.6 μm, which resulted in a Young’s modulus and tensile strength of 67–77 GPa and 1030–1170 MPa, respectively.^17^ This suggests that the tensile properties can be improved by reducing the fiber diameter, which can be done in the dry-jet wet spinning process by changing the DR or diameter of the spinneret capillaries.

The tensile properties of the CFs in this work are lower than those of commercial standard modulus PAN-based CFs, which typically have a Young’s modulus and tensile strength of 200–300 GPa and 3000–6000 MPa, respectively.^2^ This was however expected since cellulose-based CFs require hot stretching at >2000 °C (graphitization) to develop an ordered graphite structure that can give high-performance CFs with a Young’s modulus up to 500 GPa.^4,6,7^ In the future, the effect of applying higher carbonization temperatures during continuous conversion and its impact on the CF tensile properties and structure will be investigated.

### Fiber Morphology of PFs and CFs Obtained by Continuous Conversion

**Figure 4:**
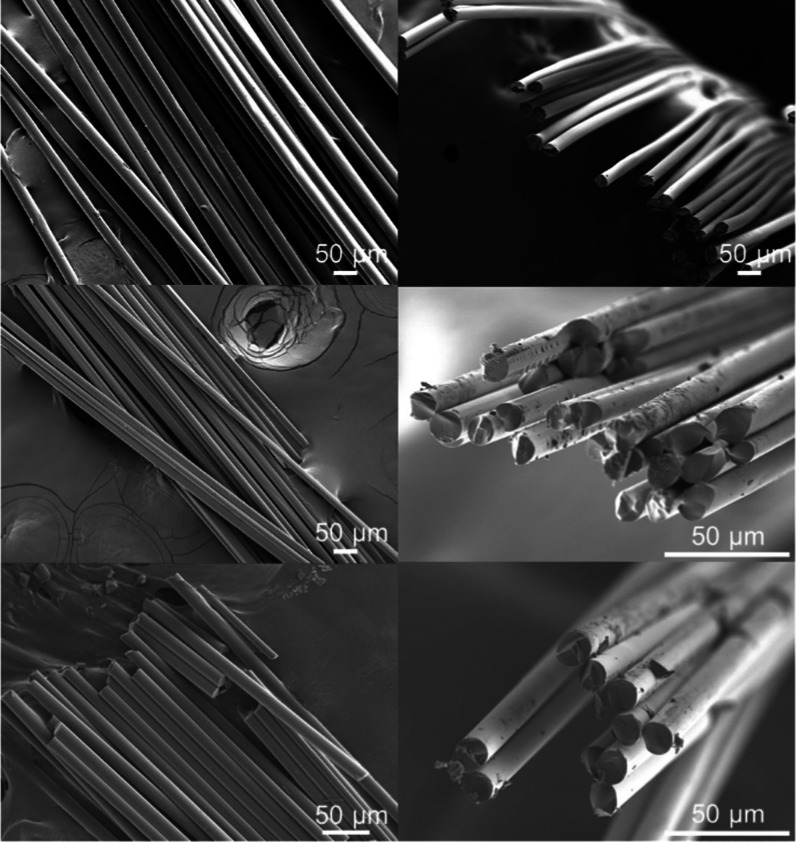
SEM images of the surface (left) and cross section (right) of the DR2 lignin–cellulose PF (top) and of the CFs derived via continuous conversion using a carbonization temperature of 800 °C (middle) or 1000 °C (bottom).

The fiber morphology can influence the tensile properties of the CF and the adhesion of the CFs to the matrix in a fiber-reinforced composite. Figure 4 shows the surface and cross-sectional morphology of the DR2 PFs and of the resultant CFs derived at 800 and 1000 °C. As expected, the morphology is preserved after the conversion to CF. The fibers have a circular and solid cross section and a smooth surface, in agreement with previous observations during the conversion of lignin–cellulose PFs.^16,19^ This contrasts to the recent findings of Le et al., who obtained hollow CFs after continuous conversion of hardwood organosolv lignin–cellulose PFs.^19^ The different CF morphology obtained in the present work is attributed to the use of softwood lignin instead of hardwood lignin, as the former has a higher thermal reactivity, which results in the formation of less volatiles that may result in detrimental voids during conversion.^8,9,23^

The CFs in the present work were partially separable by hand. Some fiber–fiber joints in the PF tow were observed (Figure S3), and these were preserved after conversion into CF, suggesting that a different spin finish and/or application is necessary.^37^ This phenomenon has also been observed for cellulosic rayon and lyocell fibers, indicating that the spinning process itself, e.g., the washing and drying conditions, influences the PF quality.^38^

In this work, online washing and drying during the fiber spinning were used, and the PF tow was easier to separate than in our previous work, where the wet PF was wound on a bobbin and washed and dried separately after the spinning.^16−18^ To minimize fiber fusion, it is wise to select the conversion conditions (e.g., temperature profile and gas flow) that minimize recondensation of tarry volatiles on the fiber surfaces, which can cause fiber stickiness.^6^ However, this was likely not a problem in the present work since the PF tow was small and the gas in the furnace was exchanged about five times per min, based on the furnace volume (1.4 L), the gas flow set (4 L/min), and the stabilization temperature of 275 °C. Residual solvent (EMIMAc) in the PF may also lead to fiber stickiness and increase the fusion during conversion. The CHN analysis of the PFs revealed that the residual EMIMAc was about 7 wt % in the DR2 fibers and 3 wt % in the DR4 fibers (Table S4). The difference in the residual solvent of the PFs is probably related to the difference in fiber diameter (Table S1). In summary, these results show that the fiber morphology is similar to that obtained during batchwise conversion^17,18^ and that more work is needed to optimize the PF quality, including washing, drying, and spin finishing.

### Effect of Stabilization Time during Continuous Conversion

Stabilization is the most time-consuming conversion step, and it is therefore a bottleneck in CF manufacturing.^22^ The prime goal of stabilization is to convert the PF into a fiber that can undergo carbonization without giving fused fibers or loss of shape. The stabilization conditions can also influence the tensile properties and the yield of the final CF. The residence time (30, 45, and 60 min) during the stabilization of the DR2 PFs was studied with regard to its impact on the chemical changes and the tensile properties of both the stabilized PF and the final CF. TGA was also used to monitor the effect of stabilization time on thermal behavior.

**Figure 5:**
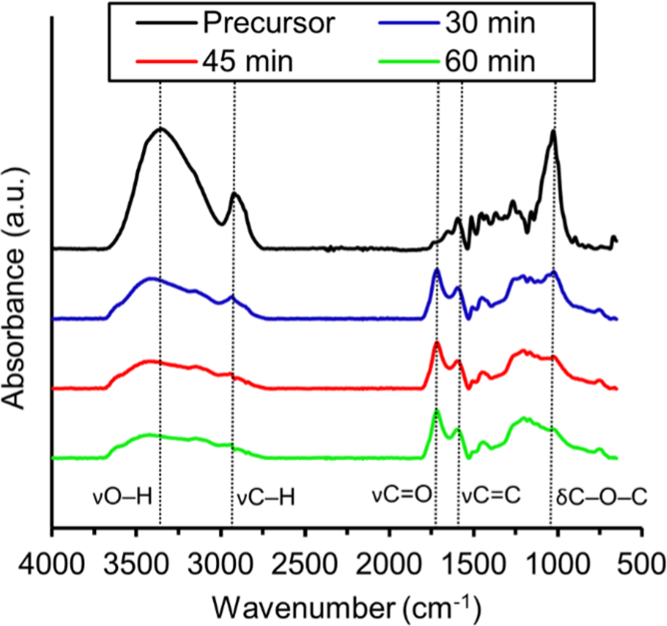
FTIR spectra of the lignin–cellulose PFs (DR2) before and after continuous stabilization using residence times of 30, 45, and 60 min. The broken vertical lines show the assigned functional groups.

Figure 5 shows FTIR spectra of the PF before and after stabilization at different residence times in the furnace. The relative intensity of the O–H band around 3300 cm^–1^ decreased with increasing stabilization time, whereas the intensity of the carbonyl band (C=O) around 1716 cm^–1^ increased. This reflects the oxidation of the hydroxyl groups in both softwood kraft lignin and cellulose and is the most typical stabilization reaction.^17,21^ In addition, the intensity of aromatic signals, e.g., that at 1600 cm^–1^ (C=C), increases relative to the intensity of the aliphatic C–H signal at 2940 cm^–1^. This means that the fibers, after stabilization, have a more dehydrogenated structure with more aromatic moieties, which explains why brown lignin–cellulose PFs turn black after stabilization.^39^ Furthermore, the FTIR spectra show a decrease in relative intensity of the C–O–C signal at 1020 cm^–1^, originating from scission of bonds in softwood kraft lignin and cellulose, e.g., depolymerization and ring-opening of the latter. Overall, the FTIR results for PFs stabilized continuously and batchwise are in good agreement.^17^

**Figure 6:**
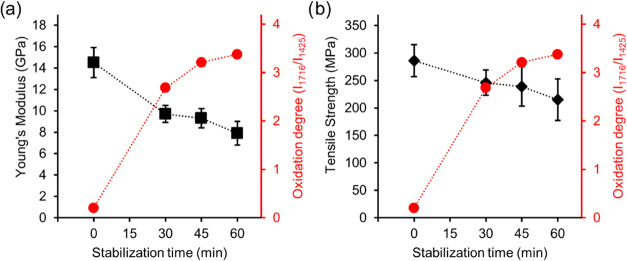
Effect of residence time during continuous stabilization on (a) Young’s modulus and (b) tensile strength of lignin–cellulose PFs (DR2). The degree of oxidation calculated from the FTIR spectra (Figure 5) is also shown. The error bars are standard deviations.

Oxidation is one of the major reactions during stabilization. The degree of oxidation can be semi-quantitatively calculated from the ratio of the intensity of the carbonyl band (1716 cm^–1^) to that of the signal with the smallest change, which is the C–H signal at 1425 cm^–1^.^17,21^Figure 6 shows the tensile properties and the degree of oxidation as functions of the stabilization time during continuous conversion. Both the Young’s modulus and the tensile strength decreased with increasing stabilization time, whereas the degree of oxidation increased. The greatest decrease in tensile properties and the greatest increase in the degree of oxidation were observed during stabilization for 30 min, indicating that the stabilization was more efficient during the first 30 min. The stabilization leads to depolymerization of cellulose (see 1020 cm^–1^ in Figure 5) and destruction of its crystalline structure, which lowers the tensile properties of the lignin–cellulose fibers. The degree of oxidation after 30 min was 2.7, which increased to 3.4 after 60 min. The degrees of oxidation in the present work were significantly higher than those obtained in our previous work on the batchwise stabilization of 70/30 wt/wt lignin–cellulose PFs, where the degree of oxidation was 3.1 after 300 min at 250 °C.^17^ These results suggest that the higher stabilization temperature used in the present work (275 °C) accelerates the stabilization reactions, in agreement with the findings of Byrne et al.^21^

The tensile strengths of the PF and the stabilized PF were about the same (Figure 6b), whereas during the stabilization of cellulose fibers (rayon), a decrease in tensile strength is observed due to depolymerization and destruction of the crystalline structure.^28^ Kraft lignin may undergo various chemical cross-linking reactions during stabilization that increases its molecular mass, and this explains why the tensile strength of melt-spun lignin fibers is constant or increases after stabilization, although the tensile strength is still lower than that of dry-jet wet spun lignin–cellulose fibers.^35^ The PFs in this work consisted of a 50/50 blend of softwood kraft lignin and cellulose pulp, and their behavior during stabilization may balance each other in terms of loss in tensile strength during stabilization. The same behavior was recently observed by Le et al., who studied the continuous conversion of lignin–cellulose PFs using hardwood organosolv lignin.^19^ This suggests that it is beneficial to coprocess softwood kraft lignin and cellulose into CF instead of processing the polymers separately.

**Figure 7:**
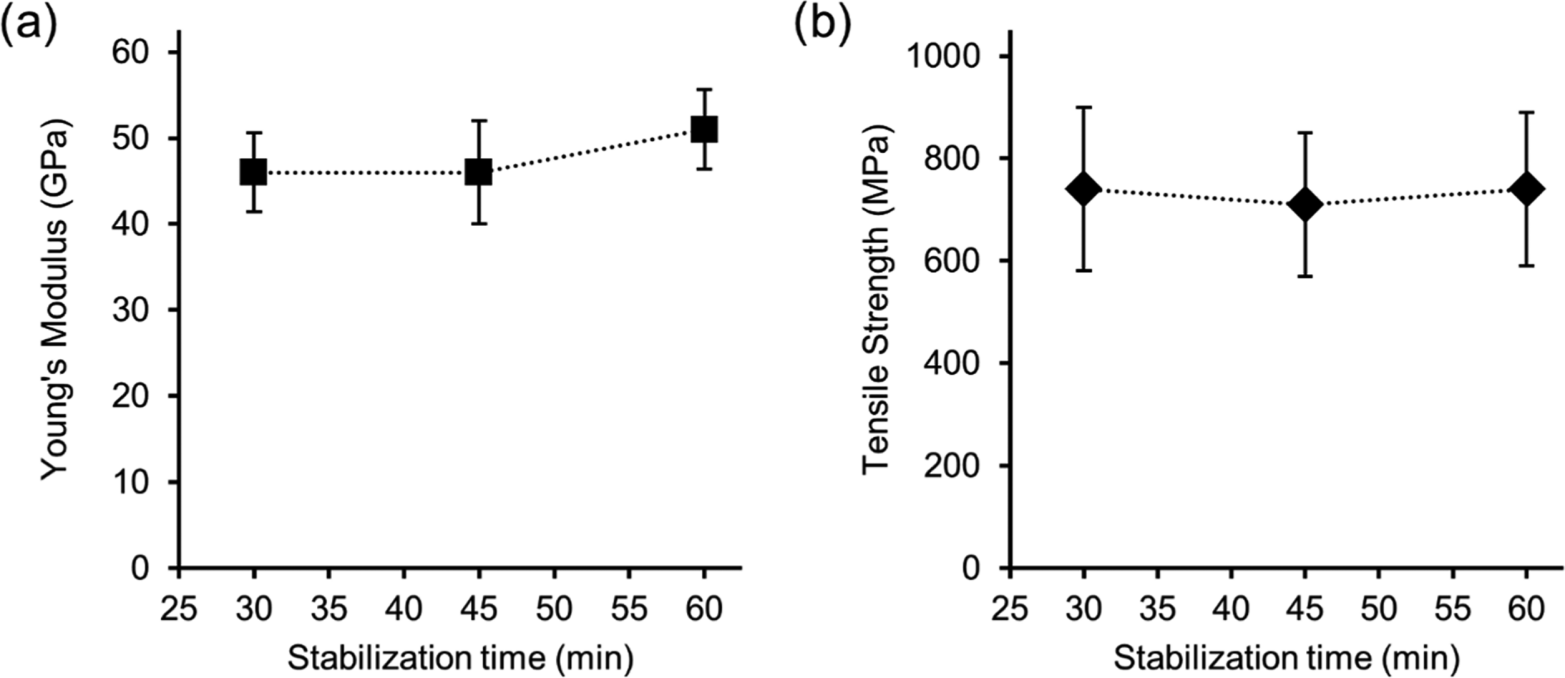
Effect of stabilization time (30, 45, and 60 min) on (a) Young’s modulus and (b) tensile strength of CFs derived from lignin–cellulose PFs (DR2). The final temperature during carbonization was 1000 °C. The error bars are standard deviations.

The PFs were stabilized for 30, 45, and 60 min and then converted to CFs using a final temperature of 1000 °C; see Table 1 for details. Figure 7 shows the Young’s modulus and tensile strength of the CFs, while a full summary of the tensile results can be found in Table S2. The CFs had a Young’s modulus and tensile strength of 46–51 GPa and 710–740 MPa, respectively, indicating that the stabilization time had no significant influence on the tensile properties. This indicates that a stabilization time of 30 min can be used, reducing the energy consumption during the conversion of lignin–cellulose PFs into CFs. This is supported by the negligible difference observed in fiber separability of the obtained CF tows, regardless of the stabilization time. Even if a stabilization time of 60 min is used, the total conversion time would be 92 min, which is comparable to the time required to produce commercial CF from PAN.^4^

The effect of stabilization time on the thermal behavior and mass yield of the fibers during stabilization (air) and carbonization (nitrogen) was examined with TGA. This is a viable approach as it requires only a few mg of material. Figure S4a–c shows TGA thermograms of the lignin–cellulose PFs (DR2) during stabilization in air for 30, 45, and 60 min, while Figure S4d shows the carbonization up to 1000 °C of the stabilized PFs, including 17 min at a constant temperature. Clearly, a longer stabilization time decreased the stabilization yield but raised the carbonization yield. The total CF yield using 30 min stabilization was 21.6 wt %, which increased to 24.1 wt % after 60 min stabilization, suggesting that a longer stabilization time increases the CF yield to some extent, in agreement with previous findings.^17,21^ However, the CF yield when using 30 min stabilization in the TGA, 21.6 wt %, was lower than the total CF yield, 29 wt %, obtained with 30 min of stabilization during the continuous conversion (Figure 2a). This difference may be explained by the longer total treatment time in the TGA, as a heating rate of only 10 °C/min was employed, compared to the “infinite” heating rate used in the continuous experiments. In addition, the gas flow conditions were different in the TGA from those in the tube furnace used for the continuous trials. To maximize the yield, a stabilization profile with a lower temperature and a longer residence time may be the best, since a temperature closer to the degradation temperature of the raw materials shortens the stabilization time but usually at the expense of the yield.^21,37^

### Interfacial Shear Strength of Epoxy Resin and Lignin–Cellulose-Derived CFs

**Figure 8:**
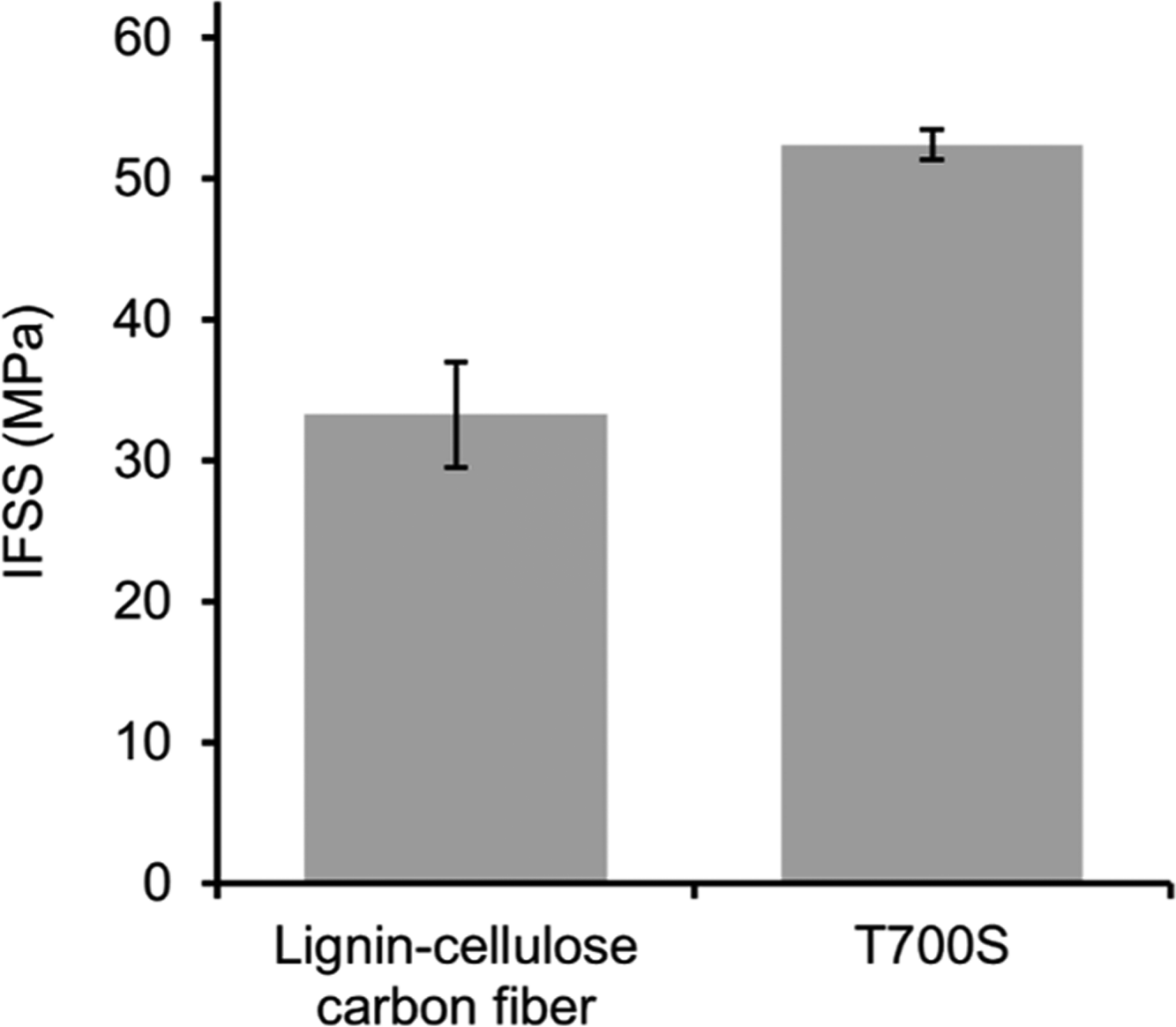
Interfacial shear strength (IFSS) of cured epoxy resin droplets on lignin–cellulose-derived CFs (DR2, stabilization time 30 min) and commercial PAN-based CFs (T700S). Error bars are the standard deviations.

Important for the mechanical performance of a fiber-reinforced composite is the creation of a strong fiber–matrix interface, but no data is available on how these lignin–cellulose-derived CFs could perform in a composite.^40^ Microbond tests that measure the interfacial shear strength (IFSS) provide information about the interfacial adhesion in a fiber–matrix system. Figure 8 shows the IFSS of cured epoxy to the lignin–cellulose CFs and, for comparison, commercially available T700S PAN-based CF. The lignin–cellulose-derived CFs had an IFSS of 33.3 MPa, which was around 37% lower than the IFSS of the T700S CF (52.4 MPa). This difference may be due to a difference in surface properties of the CFs. Commercial PAN-based CF is usually surface-treated by, e.g., plasma or electrolytic oxidation to improve the CF–epoxy adhesion. This type of treatment can increase the IFSS by 10–200%, depending on the method used.^4^ Although the IFSS was lower than that of the reference, it was still in the same range as epoxy–aramid (29.8–54.2 MPa) but lower than epoxy-glass fiber (52.7 MPa), measured with the same device.^27,41^

## Conclusions

This work demonstrates the successful continuous conversion of dry-jet wet spun lignin–cellulose PFs (50/50 wt/wt) into CFs using softwood kraft lignin and cellulose pulp as sources.

The cross section of the CFs was circular and without large pores, in contrast to other published work.^19^ CFs were prepared at 800 or 1000 °C using industrially relevant conversion times (1.0–1.5 h), comparable to the conversion of commercial fossil-based PAN into CF. Continuous stabilization for 0.5–1.0 h at 275 °C had no significant impact on the CF tensile properties, yield, and fiber separability. A carbonization temperature of 1000 °C instead of 800 °C gave higher CF tensile properties.

The CFs obtained after continuous carbonization at 1000 °C and a stabilization time of 0.5 h had a conversion yield of 29–30 wt %. Regardless of conversion time and CF diameter (12–15 μm), the CFs derived at 1000 °C had a Young’s modulus of 46–51 GPa and a tensile strength of 710–920 MPa, suggesting that they can be classified as general performance CFs for composites.

In comparison, CFs derived via batchwise conversion (total conversion time 7 h) had a similar tensile strength (800–920 MPa) but a higher Young’s modulus (63–67 GPa) and conversion yield (34 wt %). The use of a tensiometer and a larger PF tow would enable optimization of the tension during continuous conversion, leading to improved CF tensile properties. In addition, the PFs were partially separable from the tow, suggesting that more work is needed to improve the PF quality by, e.g., finding a suitable spin finish that prevents the formation of fiber joints in the spinning process.

For the first time, the interfacial shear strength (IFSS) of lignin–cellulose-derived CFs to epoxy resin was measured, which was about 33 MPa, i.e., in the same range as the IFSS of epoxy to aramid fibers. It is suggested that measurements of IFSS can be used for the screening of suitable fiber-matrix and sizing combinations, which is useful in composite development.

## Supporting Information Available

Additional information regarding results from tensile tests, PF defects (SEM), CHN analysis, and Raman and the stabilization study using TGA (PDF)
