# Supplementary material for: Continuous Stabilization and Carbonization of a Lignin–Cellulose Precursor to Carbon Fiber
Source: ACS Omega. 2022 May 5;7(19):16793–802. doi: 10.1021/acsomega.2c01806 (PMC9118265; doi:10.1021/acsomega.2c01806)
Supplement: Supplementary file 1 — ao2c01806_si_001.pdf [file ao2c01806_si_001.pdf]

Supporting information

Continuous Stabilization and Carbonization of a Lignin-Cellulose Precursor to Carbon Fiber

Andreas Bengtsson[*,†], *Jenny Bengtsson*[‡], *Kerstin Jedvert*[‡], *Markus Kakkonen*[§], *Olli Tanhuanpää*[§], *Elisabet Brännvall*[†] *and Maria Sedin*[†]

†Division Bioeconomy and Health, RISE Research Institutes of Sweden, Box 5604, SE-114 86 Stockholm, Sweden

‡Division Material and Production, RISE Research Institutes of Sweden, Box 104, SE-431 22 Mölndal, Sweden

[§]Fibrobotics OY, Korkeakoulunkatu 1, FI-33720 Tampere, Finland

*Corresponding author E-mail: andreas.bengsson@ri.se

Number of Pages: 5

Number of Figures: 4 (Figures S1–S4)

Number of Tables: 4 (Tables S1–S4)

Figures

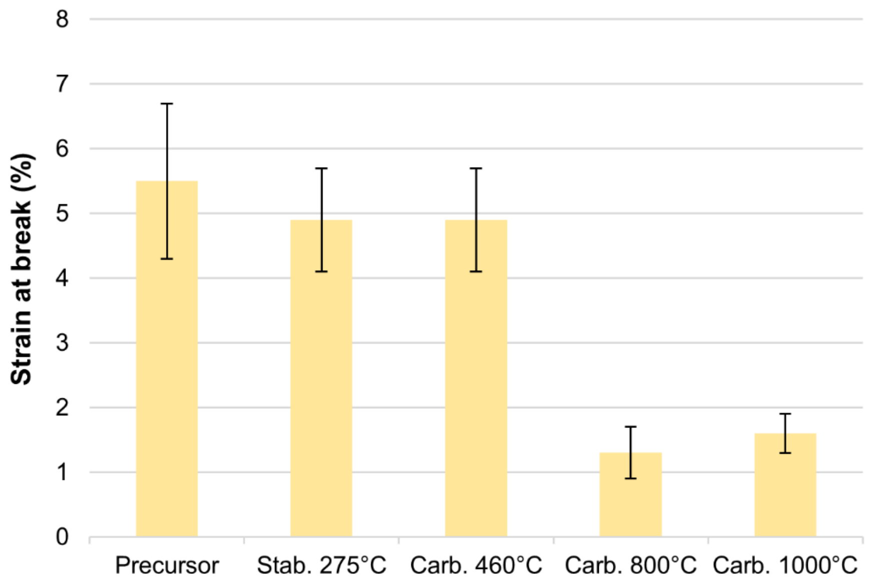

Figure S1. Effect of treatment temperature on the strain at break from tensile tests upon preparation of carbon fibers derived via continuous conversion of the lignin-cellulose precursor spun with DR 2 using a stabilization time of 30 min and a final carbonization temperature of 1000 °C. The error bars represent the standard deviation.

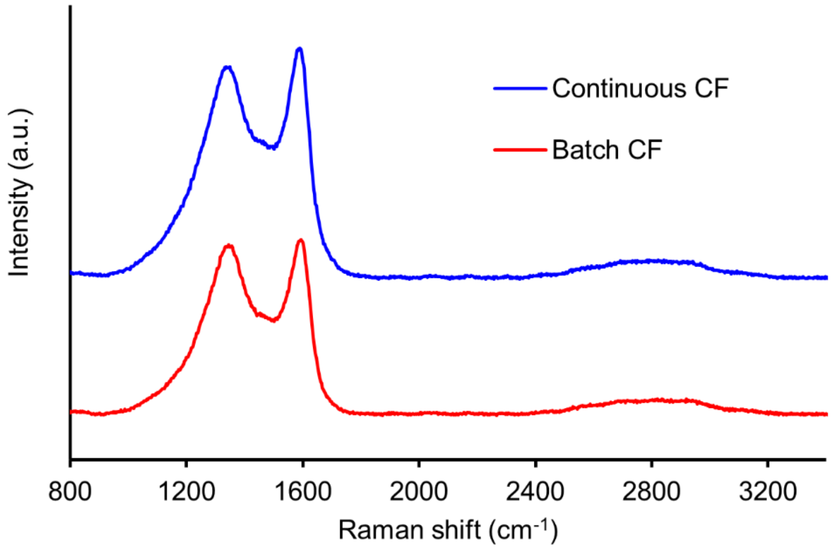

Figure S2. Raman spectra of CFs derived at 1000 °C via batch-wise and continuous conversion using the DR 2 lignin-cellulose precursor and a stabilization time of 30 min.

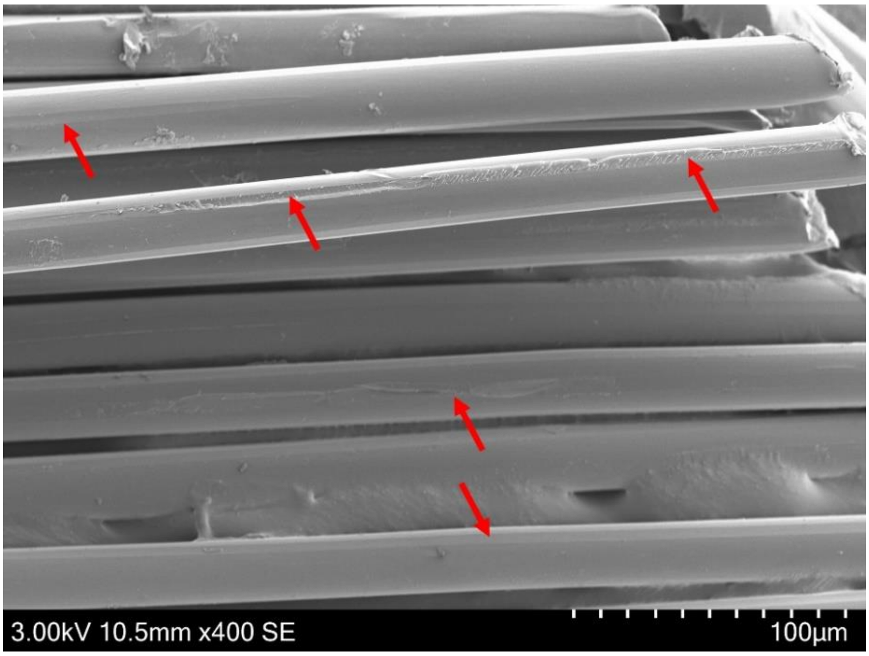

Figure S3. SEM image showing defects on the lignin-cellulose precursor (DR 2) due to joints formed between individual filaments during the precursor spinning.

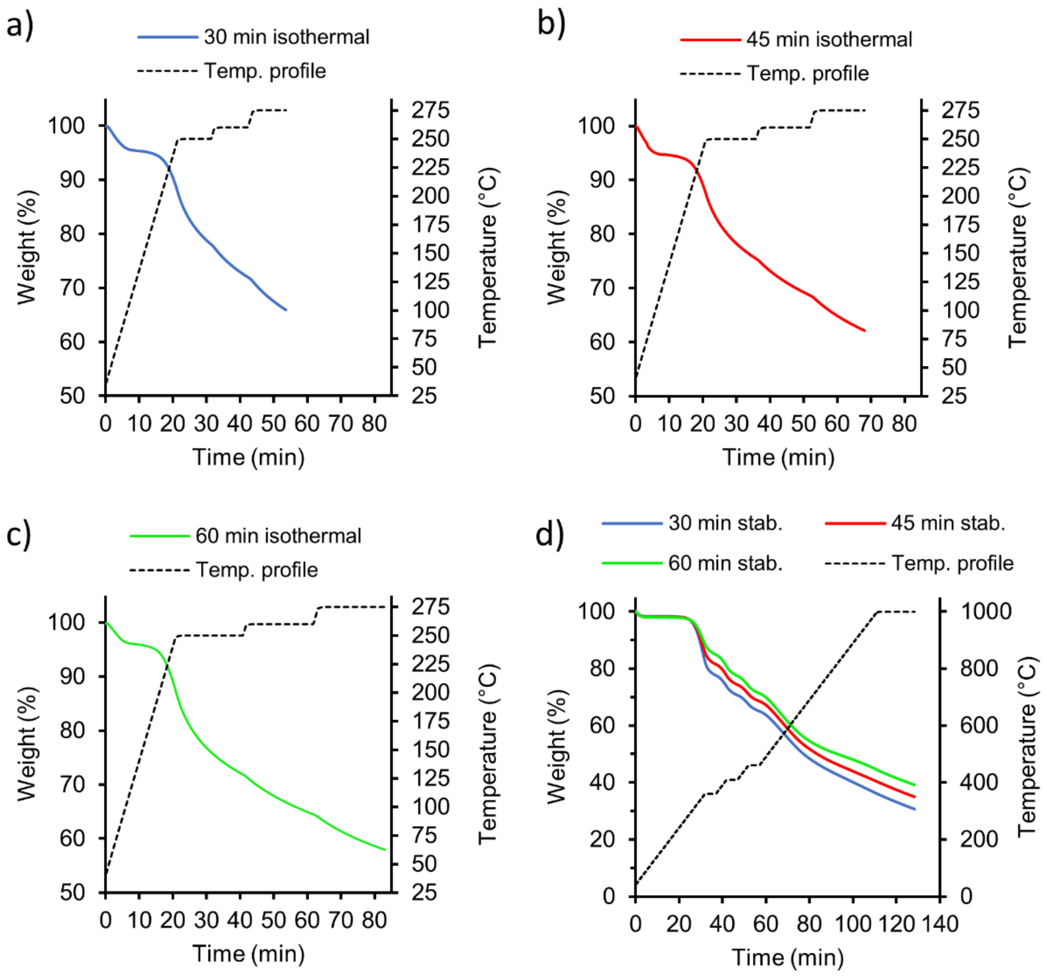

Figure S4. TGA of lignin-cellulose precursors fibers (DR 2) mimicking the stabilization and carbonization profile during continuous conversion trials (see Table 1). Isothermal times during stabilization (air) at 250, 260 and 275 °C: a) 30 min, b) 45 min and c) 60 min. The carbonization (nitrogen) of the stabilized fibers is shown in d) using 5 min isothermals at 350, 410 and 460 °C and a 17 min isothermal at 1000 °C. The heating rate in all TGA-runs were 10 °C/min.

Tables

Table S1. Tensile properties of lignin-cellulose precursors with different draw ratios (DR).

Precursor	Diameter (µm)	Elongation at break (%)	Young's modulus (GPa)	Tensile strength (MPa)
DR 2	27.9 (2.1)	5.5 (1.2)	14.5 (1.4)	290 (30)
DR 4	23.3 (1.9)	9.7 (2.3)	13.1 (1.6)	310 (40)

Table S2. Tensile properties of carbon fibers derived from the lignin-cellulose precursor fibers (DR 2 and 4) via continuous or batch-wise conversion. The final temperature during carbonization was 1000 °C. The values in bracket is the standard deviation.

Precursor	Conversion type	Stabilization time (min)	Young's Modulus (GPa)	Tensile Strength (MPa)	Strain (%)	Diameter (µm)
DR2	Continuous	30	46 (4.6)	740 (160)	1.6 (0.3)	14.5 (1.1)
DR2	Continuous	45	46 (6.0)	710 (140)	1.6 (0.3)	14.8 (1.0)
DR2	Continuous	60	51 (4.6)	740 (150)	1.5 (0.3)	15.3 (0.8)
DR4	Continuous	30	49 (6.9)	840 (190)	1.7 (0.3)	11.9 (1.2)
DR2	Batch	30	63 (5.3)	800 (170)	1.3 (0.3)	15.3 (0.9)
DR4	Batch	30	67 (10.5)	920 (200)	1.4 (0.4)	12.0 (1.0)

Table S3. Calculated data from high-resolution Raman spectra (1800 gratings/mm) of CFs derived at 1000 °C via batch-wise and continuous conversion. The full width at half maximum (FWHM) and intensity ratio of the D and G band (I_D/I_G) is presented. The value in brackets is the standard deviation.

Sample	$FWHM_D$ (cm^{-1})	$FWHM_G$ (cm^{-1})	I_D/I_G
Continuous	239 (3.8)	106 (4.8)	0.88 (0.01)
Batch	224 (9.4)	103 (4.8)	0.85 (0.01)

Table S4. Elemental composition from CHN-analysis of lignin-cellulose precursors spun with different draw ratios. The EMIMAc content was calculated based on the nitrogen content of the fibers, corrected for the nitrogen content present in the softwood kraft lignin. The oxygen content (%) is obtained by calculating the difference from the CHN-analysis (100-[C+H+N]).

Precursor	C (%)	H (%)	N (%)	O (%)	EMIMAc (%)
DR 2	52.2	6.2	1.2	40.4	7
DR 4	52	5.7	0.5	41.8	3
